# Visually challenging conditions on sign language intelligibility show behavioural analogies with spoken language

**DOI:** 10.1038/s41598-026-57531-0

**Published:** 2026-06-13

**Authors:** Cristina Tobías Figuerola, Carine Signoret, Emil Holmer, Josefine Andin

**Affiliations:** Linköping University, Linköping, Sweden

**Keywords:** Neuroscience, Psychology, Psychology

## Abstract

This study aimed to understand how visually degraded conditions affect the intelligibility of isolated signs in sign language, and how these conditions influence perceived difficulty. Twenty-nine fluent users of Swedish Sign Language viewed 100 isolated signs presented under two types of visual degradation: spectral degradation (pixelation) and background noise (salt-and-pepper noise), each across five levels of degradation. Participants identified each sign and rated the perceived difficulty. Generalized linear mixed models were used to evaluate the effect of level of degradation on intelligibility and perceived difficulty, separately and for each type of degradation. Psychometric functions were estimated, and generalized linear mixed models were used to analyse the relationship between intelligibility and perceived difficulty. Both types of degradation significantly reduced intelligibility and increased perceived difficulty. Psychometric curves showed that intelligibility plateaued around 70% accuracy, mirroring results from speech-in-noise research. Correct responses were consistently rated as easier to perceive, and an interaction between degradation level and response accuracy was observed only in the spectral degradation condition, suggesting distinct perceptual processing mechanisms. Visual degradations affect sign language intelligibility in ways comparable to auditory distortions in speech, with differences between degradation types suggesting distinct perceptual strategies. These findings have implications for theories about linguistic processing in perceptually challenging conditions and call for further investigation into cognitive and linguistic factors that influence sign perception in degraded conditions.

**Supplementary Information:**

The online version contains supplementary material available at 10.1038/s41598-026-57531-0.

## Introduction

Intelligibility, i.e., how much of the intended message is being understood^1^, is a key aspect of communication. Communication often occurs in perceptually challenging environments that reduce intelligibility and the cognitive mechanisms required to adapt to these environments have been thoroughly studied in speech (for a review, see^2^. Perceptually challenging conditions typically increase the cognitive effort required for effective communication, leading to discomfort, stress, and fatigue^3^. However, these effects have not been adequately studied for communication in sign language. Early studies in sign language have tried to assess intelligibility (e.g.^4^), though there is yet no standardised methodology for this type of research^5^. The present study aims to examine how visually challenging conditions affect the intelligibility of isolated signs, as well as the signers’ perceived difficulty of sign identification. We expect our findings to provide further insights into theories of language processing that suggest perceptually challenging conditions engage similar mechanisms, regardless of language modality^6,7^. Beyond theoretical contributions, our study aims to serve as a methodological reference for future research exploring sign language under perceptually challenging conditions.

In the auditory domain, listeners adapt to perceptually challenging conditions by habituation^8,9^ or by the use of technical aids that increase the signal-to-noise ratio (SNR). The relationship between signal quality and speech intelligibility has been established using psychometric intelligibility measures. Psychometric functions can be used to illustrate how performance on a behavioural task relates to specific characteristics of the stimuli. This is typically defined by two key parameters: the threshold, indicating the level of noise that can be added while still reaching a certain performance level (such as 50% accuracy), and the slope, which reflects the rate of performance improvement as SNR increases. Pavel and colleagues^4^ investigated how changes in visual contrast and noise levels influenced sign language intelligibility and defined a psychometric curve to show that signal quality impacts sign language intelligibility similarly to how auditory signal quality influences spoken language intelligibility. Following language models assuming language processing to be modality independent (e.g., ELU model^6,7^, cognitive mechanisms supporting processing in perceptually challenging conditions should be similar across modalities. Therefore, it can also be hypothesized that decreased signal quality increases the effort needed to decode messages also for sign language.

To find equivalences in how intelligibility is impacted by different types of perceptually challenging conditions across language modalities, it is necessary to understand the characteristics of the conditions both in speech (for a review, see^10^ and in sign. Two of the most studied methods for generating adverse listening conditions in the research of speech-in-noise are the addition of background noise and the degradation of the spectral quality of the signal using filtering methods. In both the auditory and the visual modalities, background noise refers to perceptual conditions in which competing stimuli interfere with the language signal, such as distracting sounds in speech or a cluttered visual environment in sign language. Background noise in the form of salt-and-pepper (S&P) noise in sign language was used by Pavel et al. to study intelligibility of individual signs in American Sign Language (ASL). They evaluated the intelligibility of a set of common ASL signs in three different SNR conditions (pure noise, 50% SNR and 75% SNR) and derived a psychometric curve that showed performance asymptotes at pure noise and 50% SNR. Spectral degradation in the auditory domain occurs when the fine spectral details of the signal are removed while preserving the temporal envelope. One method to artificially create this condition is through noise vocoding. This acoustic transformation technique achieves synthesised speech with reduced temporo-spectral details by substituting the original wideband speech signal with a variable number of noise bands. Pixelation (altering the frequencies in the spatial domain by averaging a cluster of pixels into one) has been employed in behavioural research (e.g.^11^) and to simulate artificial vision via retinal implants^12,13^, in a manner similar to how noise-vocoding is used to simulate cochlear implant hearing. However, to the best of our knowledge, this specific type of degradation has not been applied yet to study the effects of degradation on sign language intelligibility.

Another aspect of the study of intelligibility is the effort required to comprehend language in challenging conditions. In speech, the study of listening effort has shown that there are individual differences in how challenging it is to perceive speech in noise even when intelligibility (e.g.^14–16^) or SNR^17^ is high. Due to the lack of studies in sign language intelligibility, it is not known if these relationships generalise to this language modality.

For the purpose of this study, we have created two different perceptually challenging conditions for the visual modality based on the ones more commonly studied for speech: background noise and spectral degradation. The aim is to investigate how these conditions affect sign language intelligibility and their relationship with perceived difficulty. To characterise how intelligibility change with different levels of degradation, we will establish a psychometric curve for both conditions. Additionally, we seek to understand how intelligibility is related to perceived difficulty in sign language and how this relationship interacts with the level of degradation. We expect that, for both conditions, a reduced SNR will correspond to decreased intelligibility and increased perceived difficulty. We expect our findings to provide further insights into theories of language processing that suggest that perceptually challenging conditions engage similar mechanisms, regardless of language modality^6,7^. Beyond theoretical contributions, our study aims to serve as a methodological reference for future research exploring sign language under perceptually challenging conditions. From a societal perspective, this work is relevant as it addresses the universal experience of language processing in difficult perceptual environments. By understanding the cognitive demands of such conditions, we can make informed adjustments to learning environments in schools, workplaces, and social settings, as well as address the challenges faced by those with hearing or vision loss.

## Methods

### Participants

Our final dataset consisted of 29 participants aged 18–45 years (m = 36, sd = 7; 6 deaf; 5 male and one with undefined gender), all of whom were self-reportedly fluent in Swedish Sign Language (*Svenskt teckenspråk*; STS). Participants were selected according to predefined inclusion and exclusion criteria to ensure the consistency and validity of the data. Eligible individuals were between the ages of 18–45, and self-described as a frequent user of, and fluent in, STS. Deaf participants used STS as their main mode of communication, and hearing participants were sign language interpreters and used STS for work. Moreover, they also needed to have completed upper secondary education, or equivalent. Individuals were excluded if they had a developmental, language, mental or neuropsychiatric disability, or if they were motorically limited to give a response by pressing a button. Screening was conducted via an online questionnaire to ensure adherence to these criteria.

We recruited 42 individuals who were either Deaf or who worked as STS interpreters. The participants were recruited through flyers on different social media, by contacting sign language interpreting organizations and by recommendation of previous participants. Due to age restrictions (< 46), three were not eligible for the task. Another two participants withdrew their consent, and one participant could not complete the task due to a technical issue. Among those who undertook the task, responses from five participants were not saved on the test platform. Furthermore, to be included in the analyses, a performance of at least 50% was required, which resulted in the exclusion of two additional participants. The present study was preregistered on OSF^18^.

### Ethics statement

The ethics application for the present study was approved by the Swedish Ethical Review Authority (Dnr: 2022-06463-01). This study was conducted in accordance with the General Data Protection Regulation (EU) 2016/679 (GDPR)^19^ and the principles of the Declaration of Helsinki^20^. Participants gave their written informed consent and were compensated 500 SEK (approximately 50 USD) for participating in the study.

### Materials

Isolated STS signs (*N* = 100) were selected from the STS video database developed by Witte et al.^21^. The videos were filmed using a Sony FX3 with a Tamron lens (28–75 mm f/2.8 DI III VXD G2), and quality was set to XAVZ S 4 K 100p 4:2:2 10bit. Raw videos were down-sampled to a resolution of 1440 × 1080 pixels using a H264-MPEG-4 AVC codec. The signs were performed by four actors, two females and two males, to account for the gender effects, and without lip movement or other facial expression. All signs had the same starting and final positions (hands folded). The signs were displayed at approximately two meters’ distance from the camera.

The 100 signs used in the present study (25 from each actor) were selected based on behavioural performance in a lexical decision task^22,23^, and had an identification accuracy of at least 87.5% (see supplementary Table 1 for lexical properties and meanings of chosen signs, supplementary Figs. 1, 2 and 3 for distributions of lexical frequency, phonotactic probability and neighbourhood density of the signs, respectively). For the present study, the videos were manipulated to create two types of perceptually challenging conditions: spectral degradation and background noise. The manipulated videos were then compressed with FFmpeg^24^ using a constant rate factor of 40.

#### Spectral degradation

We based our method for spectral degradation on the noise vocoding technique, which has been used for speech in previous studies. This technique involves dividing the temporal frequency of the signal using logarithmically equally spaced limits. The frequencies within those limits are averaged and referred to as bands. When creating the bands, the envelope of the signal is maintained while the temporal fine structure of the signal is averaged. To recover the signal, we created a carrier (e.g., a signal of white noise) which was convolved with the created envelope. The higher the number of bands, the more precise the reconstruction, meaning that fewer bands produce a signal with less spectral information, decreasing intelligibility^25^. For speech materials, this degradation method results in a distorted voice that varies in tone according to the type of carrier used.

**Fig. 1:**
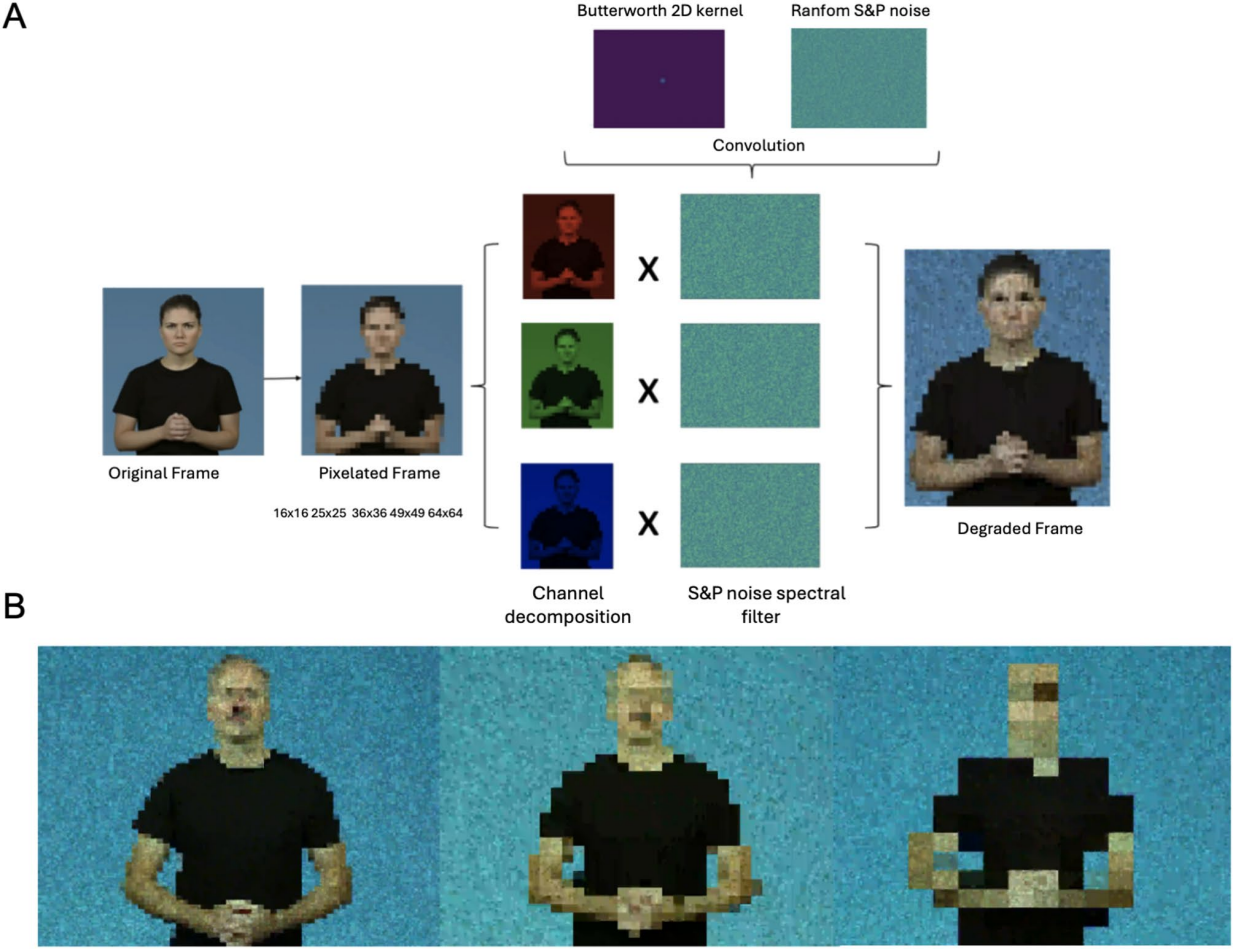
Spectral degradation process. (**A**) Each frame of the video is pixelated in 5 possible grid sizes, then a spectral filter with white noise is applied to each of the layers of the image. (**B**) Example of frames with grid sizes 64 × 64, 36 × 36,190 and 16 × 16 corresponding to levels 1, 3 and 5. Original video frames from public database (Witte et al., 2025).

To regulate the level of degradation, it is necessary to consider how information is encoded within the signal. For instance, in auditory signals, most information is contained in the temporal component, while in visual signals, it is mainly contained in the spatial component. Therefore, from our perspective, the most straightforward adaptation of noise vocoding used for auditory material in the visual domain is the pixelation of images. We implemented pixelation by segmenting each frame into a grid and calculating the average intensity of pixels within each grid cell. The number of grid cells determines the videos’ spatial resolution. The higher the number of grid cells, the closer it will be to the original spectral detail of the frame, similarly as how the number of bands determines the spectral resolution in noise vocoding^25^. As aforementioned, most of the entropy of the signal in videos is contained in the spatial domain; therefore, this is where spectral degradation must be introduced. When pixelating an image, the details of the image are lost and only the shapes of the objects remain visible (comparable to the effect achieved in noise vocoding speech). To compare with noise vocoded speech, the audio-frequency bands are translated into the number of resulting pixels by averaging the image, thus reducing the spectral spatial resolution. We created five levels of degradation that corresponded to different grid sizes. The created grids (corresponding to levels 1–5 respectively) were of 64 × 64, 49 × 49, 36 × 36, 25 × 25 and 16 × 16. The carrier is created by convolving a random white noise image with a Butterworth two-dimensional filter. The kernel-generated filter is then multiplied to each layer of every frame. The result is a pixelated image with changes in the intensity and tones of the colours (Fig. 1).

##### Note

All the figures presenting signers are from the materials used for stimuli presentation. These stimuli were recorded for a previous study^21^ and they are publicly available at http://www.osf.io/4rj5f.

#### Background noise

**Fig. 2:**
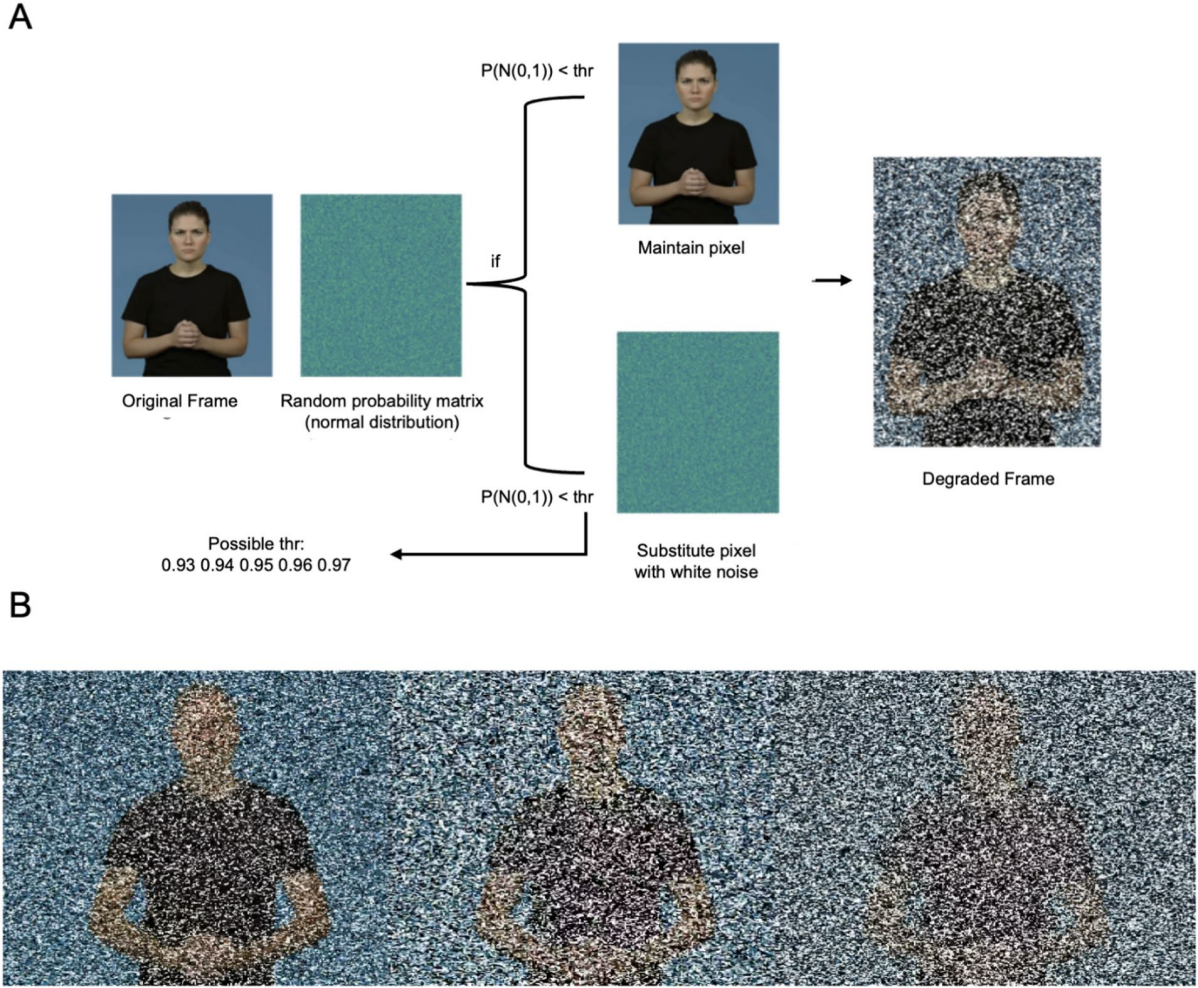
(**A**) Background degradation process. A random probability matrix is created, and the pixels of each frame are substituted accordingly by salt and pepper (S & P) noise. (**B**) Example of frames with thresholds 0.93, 0.95, 0.97. Original video frames from public database (Witte et al., 2025).

Background noise, in speech, can be created by overlaying a random white-noise signal onto the original signal, causing only the parts with higher intensity than the random signal to be distinguishable. To create background noise in the visual domain, we generated a random black and white image for each frame. This image was used to replace a certain proportion of the original pixels, creating a visual effect showing the noise image overlaying the original frame. To create a noticeable noise, we replaced pixels in groups of 4 × 4 (since one-pixel changes in a 1000 × 1000 pixel resolution are not visible to the naked eye). Since there is no standardised metric for objective video quality, we have reported the percentage of pixels that have been maintained (3–7%, levels 5 − 1 respectively) by the random noise frame instead of, e.g., the pSNR (Fig. 2).

### Task

The experiment was administered remotely via the platform Pavlovia^26^ and programmed with PsychoPy 2023.1.0^27^. A practice task was first presented with four videos (at the highest and lowest levels of degradation for both conditions to get them to know the most extreme conditions). The real task consisted of 100 videos of signs from STS with different types of noise at different levels in four blocks of 25 videos. All blocks contained both conditions and all levels of degradation. The participants were instructed to, after each video, write what they thought was being signed and to rate how difficult it was for them to see the signs. They were allowed to take breaks between blocks, but no participants needed them. Moreover, they were also informed that several translations could be possible and that it was possible to translate the signs using more than one word (e.g., an expression). Descriptions of the movement of the sign were not considered a correct answer. For the rating of the perceived difficulty, a visual analogue scale (VAS) ranging from “very easy” to “very difficult” was used (see^28^ for a similar approach in a study speech-in-noise. The dependent variables from the task was the response accuracy (0 = incorrect, 1 = correct), and the participants’ average rated difficulty from 0 (corresponding to a rating of very easy) to 10 (corresponding to very difficult).

All participants viewed the same signs without any repeated elements. The order of the degradation type and level of each sign was counterbalanced across participants. The presentation order of the signs within blocks was fixed and then reversed after half of the participants were tested to mitigate potential order effects.

### Procedure

Before conducting the task, participants filled a form with background questions. If they were eligible for the study, they met with the first author through Zoom and were given instructions on how to perform the task. Participants were allowed to take breaks between the blocks, though no participant required it. During the experiment, the camera was turned off to prevent distractions for all hearing participants. Deaf participants kept the camera on to be able to communicate with the researcher if any issue occurred. Participants were allowed to ask questions after the practice task and between the blocks. The researcher would only activate the microphone or camera during the procedure to answer participants questions or if technical issues (e.g., unvoluntary interruption of the experiment) arised.

### Statistical analyses

#### Main analyses

The main analyses consisted of the estimation of the psychometric curve with the use of scipy 1.10.1^29^ (in python 3.10^30^) and the assessment of statistical differences in intelligibility across degradation levels with generalized linear mixed models (GLMMs) using the glmmTMB^31^ package in R 2024.12.0. In our pre-registered protocol, we planned to assess statistical differences across degradation levels with an ANOVA. However, this pipeline would not allow us to control for factors such as the trial number, which might be relevant if the task performance changes as the experiment progresses.

To extrapolate the theoretical levels of intelligibility from our results, a psychometric function was estimated. The psychometric curve coefficients were estimated taking as reference points the means of intelligibility of each level of degradation in each type of perceptually challenging condition. Following descriptions of intelligibility psychometric functions in speech^32^, we modelled intelligibility using the sigmoidal function described in Eq. (1):

1 $$\:f\left(x\right)=\frac{100}{1+{e}^{m\times\:(x-c)}}$$

Where $\:m$ and $\:c$ are constants, $\:c$ is the point where intelligibility reaches 50% and $\:m$ is the slope of the function at $\:x=c$. For the spectral degradation condition, the curve was fitted using the number of pixels in each dimension (16, 25, 36, 49, 64) as the independent variable. For the background noise condition, the percentage of pixels not replaced by salt and pepper noise (7, 6, 5, 4, 3) served as the independent variable. In both conditions, the average intelligibility at these points was used as the dependent variable.

The effect of the level of degradation was estimated separately for background noise and spectral degradation. Thus, two GLMMs were used to model response accuracy (i.e., whether the written response of which sign that was presented in the video was correct, as a factor with two levels: “incorrect” = 0, and “correct” = 1) as the dependent variable. We applied a binomial distribution to analyse the effect of trial number, level of degradation, perceived difficulty and the two-way interaction between level of degradation and perceived difficulty for each type of degradation. Random intercepts and slopes for participant to account for variability. An FDR correction was applied to the GLMMs to account for multiple comparisons.

#### Exploratory analyses

Exploratory analyses were performed in R 2024.12.0 to further investigate the associations between level of degradation, response accuracy and perceived difficulty. We performed a set of exploratory analyses with GLMMs to model perceived difficulty as the dependent variable. We applied a beta distribution to analyse the effect of the level of degradation and response accuracy on perceived difficulty for each type of degradation. Difficulty ratings were scaled between 0 and 1 before the analyses to align with the assumptions of the beta family. Random intercepts and slopes by participant and random intercepts for sign ID were included to account for individual and item-level variability. The fixed effects included the trial number (an integer, from 0 to 99), level of degradation (an integer, 1 = low, to 5 = high), and response accuracy (i.e., whether the written response of which sign that was presented in the video was correct, as a factor with two levels: “incorrect” = 0, and “correct” = 1), and the two-way interaction. Statistical significance was determined using a threshold of *p*_*BH*_ < 0.05. An FDR correction was applied to the GLMMs to account for multiple comparisons.

## Results

### Spectral degradation

To examine the relationship between number of pixels per dimension (x) and intelligibility f(x), we fitted a sigmoidal function to the data. The resulting equation is described in Eq. (2):

2 $$\:f\left(x\right)=\frac{100}{1+{e}^{-0.061\times\:(x-42.495)}}$$

**Fig. 3:**
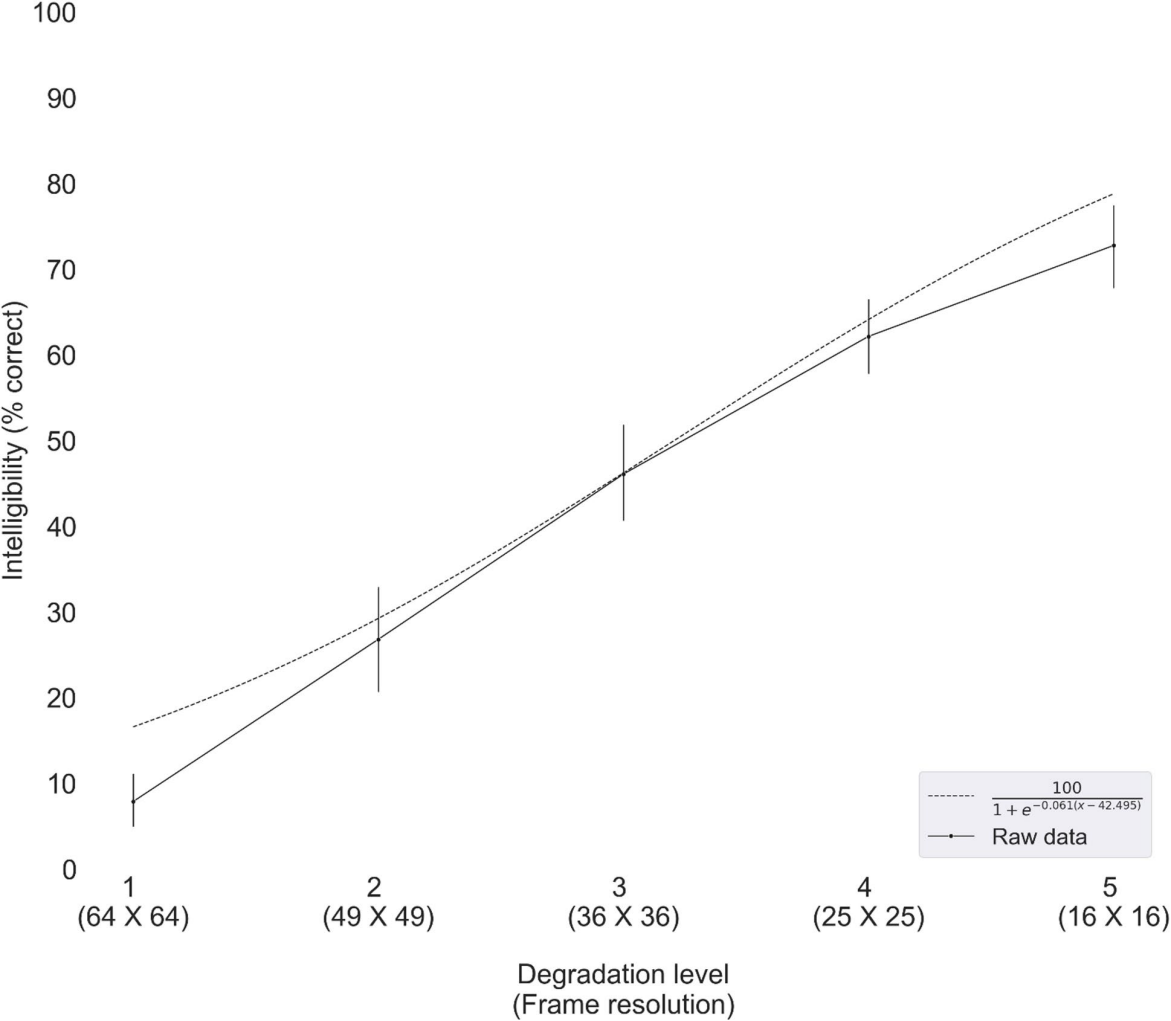
Theoretical psychometric curve (dashed) and intelligibility scores for each degradation level for spectral degradation. Error bars represent 95% confidence intervals.

The equation indicated that the inflexion point in intelligibility is reached between levels 2 and 3 ($\:c=42.495$) and that the maximum intelligibility increase is approximately 1.5% ($\:m=-0.061$) when a pixel is added to the resolution in each dimension. As seen in Fig. 3, the fitted theoretical function models the intermediate levels (2–5) accurately but overestimates the intelligibility levels close to the most extreme levels (see Fig. 3). The model fit resulted in an $\:{R}^{2}=0.94$, indicating that the sigmoidal function explains 94% of the variance in intelligibility and an $\:RMSE=5.57$, indicating that the results from the fit differ 5.57% from the original data.

The GLMM analysis showed that degradation level had a significant effect on response accuracy (Table 1), $\:\beta\:$ = 0.32, SE = 0.07, z =-5.54, *p*_*BH*_ < 0.001, showing that higher degradation levels were associated with a greater likelihood of an incorrect written response. Perceived difficulty was also associated with incorrect responses$\:,\:\beta\:$ = 0.19, SE = 0.05, z =-5.92, *p*_*BH*_ < 0.001. Furthermore, trial number was a positive predictor, showing that performance improved as the experiment progressed, $\:\beta\:$ = 1.26, SE = 0.14, z = 2.19, *p*_*BH*_ = 0.036.

**Table 1:** Results for the general linear mixed model with accuracy as outcome variable for spectral degradation. All reported metrics are standardized.

	β	Std. Error	CI	Z-statistic	Pr_BH_(>|z|)
(Intercept)	1.38	0.38	0.79–2.38	1.14	< 0.001
Trial number	1.26	0.14	1.03–1.56	2.19	0.036
Perceived difficulty	0.19	0.05	0.11–0.33	-5.92	< 0.001
Degradation level	0.32	0.07	0.21–0.48	-5.54	< 0.001
Degradation level: Perceived difficulty	1.62	0.34	1.01–1.18	2.26	0.033
Random Effects					
σ^2^	3.29				
τ_00_	0.62 ID_participant_				
ICC	0.16				
N	28 ID_participant_				
Observations	1390				
Marginal R^2^ / Conditional R^2^	0.622 / 0.682				

**Fig. 4:**
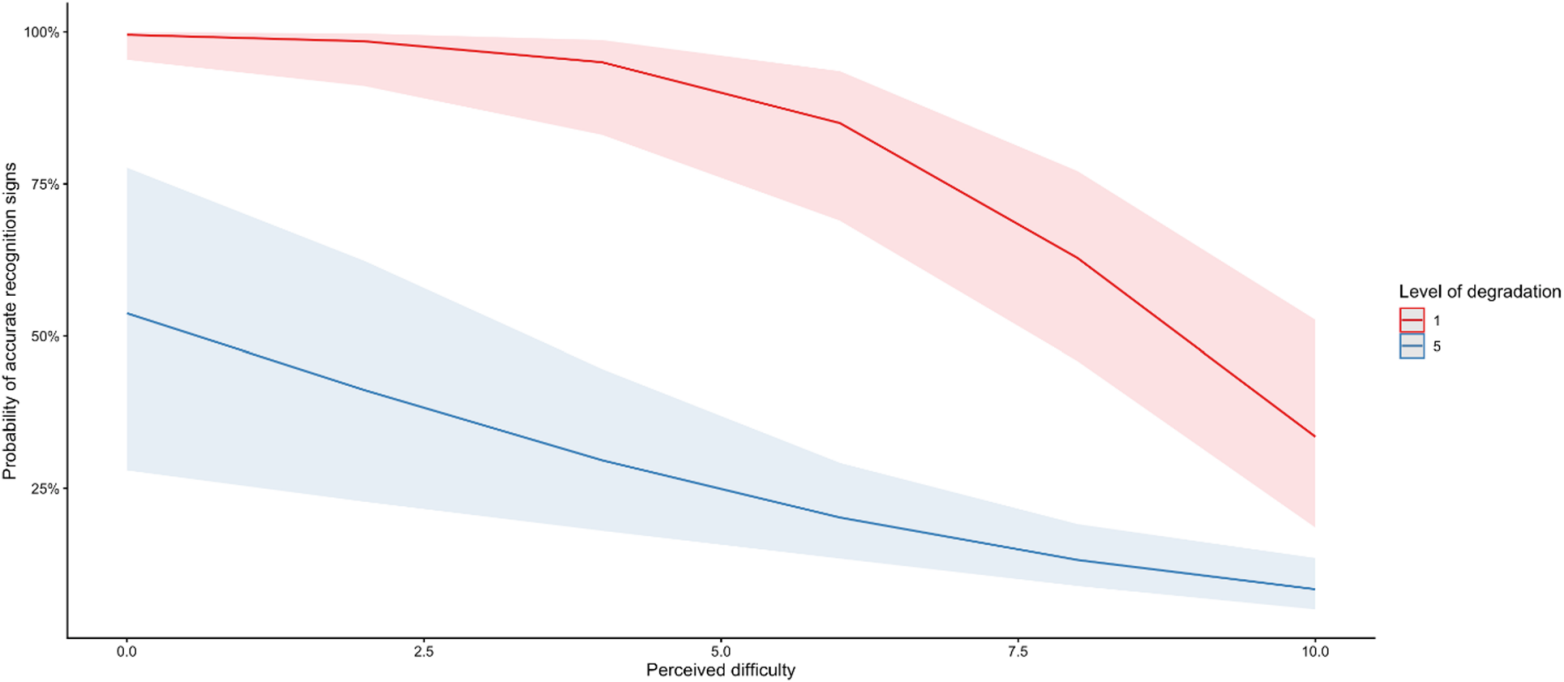
Probability of accurate recognition in background noise by perceived difficulty in levels 1 (red) and 5 (blue).

There was a significant interaction between degradation level and perceived difficulty $\:\beta\:\:$= 1.62, SE = 0.34, z = 2.26, *p*_*BH*_ = 0.033. The pattern of the interaction indicated that, as the degradation level increased, the association between perceived difficulty and correct responses is suppressed (see Fig. 4).

### Background noise

To examine the relationship between number of unreplaced pixels (x) and intelligibility f(x), we fitted a sigmoidal function to the data. The resulting equation is described in Eq. 3:

3 $$\:f\left(x\right)=\frac{100}{1+{e}^{-0.607\times\:(x-5.124)}}$$

**Fig. 5:**
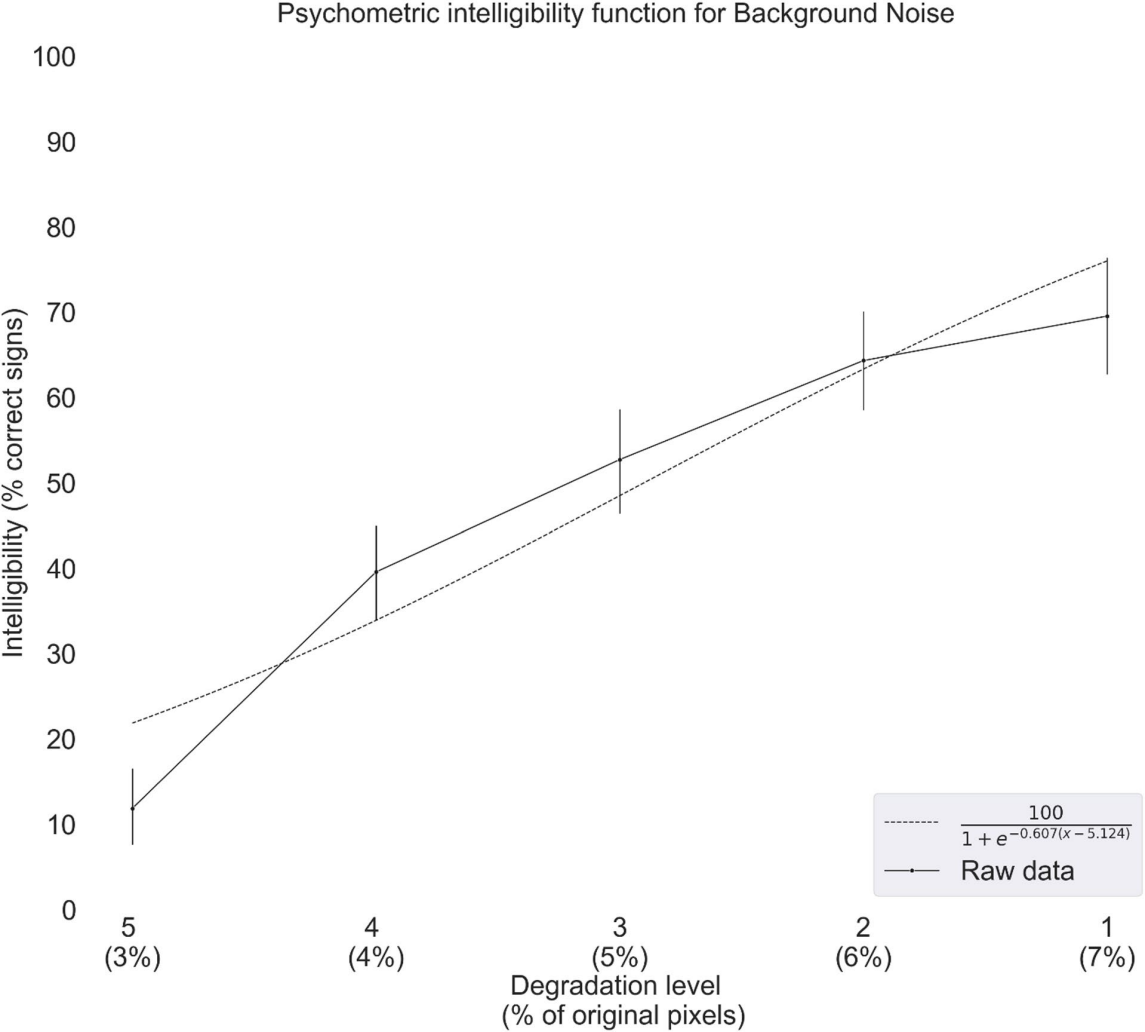
Theoretical psychometric curve (dashed) and intelligibility scores for each degradation level for background noise. Error bars represent 95% confidence intervals.

Indicating that the inflexion point in intelligibility is reached at level 3 ($\:c=5.124$) and that the maximum intelligibility increase is approximately 15% ($\:m=-0.607$) when the percentage of unreplaced pixels increases by 1. As seen in Fig. 5, the fitted curve overestimates the intelligibility at extreme levels and underestimated it in intermediate levels. The model fit resulted in an $\:{R}^{2}=0.9097$, indicating that the sigmoidal function explains 90.97% of the variance in intelligibility and an $\:RMSE=6.20$, indicating that the results from the fit differ 6.20% from the original data.

The GLMM analysis showed that degradation level had a significant effect on response accuracy (Table 2), $\:\beta\:$ = 0.41, SE = 0.08, z = -4.77, *p*_*BH*_ = 0.013, showing that higher degradation levels were associated with incorrect responses. Perceived difficulty was also associated with incorrect responses ($\:\beta\:$ = 0.18, SE = 0.05, z = -6.23, *p*_*BH*_ < 0.001). Trial number was non-significant (*p*_*BH*_ =0.545), showing that performance did not vary as the experiment progressed, $\:\beta\:$ = 1.06, SE = 0.10, z = -0.61, *p*_*BH*_ = 0.545. There was no significant interaction between degradation level and perceived difficulty, $\:\beta\:\:$= 1.24, SE = 0.24, z = 1.13, *p*_*BH*_ = 0.286.

**Table 2:** Results for the general linear mixed model with response accuracy as outcome variable for background noise. All reported metrics are standardized.

	β	Std. Error	CI	Z-statistic	Pr_BH_ (>|z|)
(Intercept)	1.72	0.52	0.95–1.80	1.80	< 0.001
Trial number	1.06	0.10	0.88–1.28	0.61	0.545
Perceived difficulty	0.18	0.05	0.11–0.31	-6.23	< 0.001
Degradation level	0.41	0.08	0.29–0.59	-4.77	0.013
Degradation level: Perceived difficulty	1.24	0.24	0.85–1.80	1.13	0.286
Random Effects					
σ^2^	3.29				
τ_00_	0.87 ID_participant_				
ICC	0.21				
N	28 ID_participant_				
Observations	1385				
Marginal R^2^ / Conditional R^2^	0.543 / 0.638				

### Exploratory analyses

#### Spectral degradation

Similar to the GLMM analyses on intelligibility, the GLMM analysis on difficulty ratings showed that degradation level had a significant effect on the difficulty ratings (Table 3), $\:\beta\:$ = 1.86, SE = 0.16, z = 7.07, *p*_*BH*_ < 0.001, showing that higher degradation levels were associated with higher perceived difficulty ratings. Items with lower intelligibility were rated as more difficult to perceive than those with higher, $\:\beta\:$ = 0.40, SE = 0.03, z = -10.89, *p*_*BH*_ < 0.001. Trial number had no significant effect on the perceived difficulty ratings ($\:\beta\:$ = 1, SE = 0.03, z = 0.10, *p*_*BH*_ =0.917).

**Table 3:** Results for the general linear mixed model with difficulty rating as outcome variable for spectral degradation. All reported metrics are standardized.

	β	Std. Error	CI	Z-statistic	Pr_BH_(>|z|)
(Intercept)	1.97	0.32	1.43–2.72	4.12	0.112
Trial number	0.95	0.03	0.90–1.02	-1.44	0.187
Degradation level	1.72	0.13	1.48–2.00	7.17	< 0.001
Response accuracy	0.38	0.03	0.32–0.45	-11.88	< 0.001
Degradation level: Response accuracy	0.92	0.07	0.80–1.07	-1.10	0.310
Random Effects					
σ^2^	1.99				
τ_00_	0.78 ID_participant_				
	0.11 ID_sign_				
τ_11_	0.05 ID_participant.Degradation level_				
ϱ_01_	-0.49 ID_participant_				
ICC	0.30				
N	28 ID_participant_				
	100 ID_sign_				
Observations	1385				
Marginal R^2 / Conditional R^2	0.194 / 0.438				

**Fig. 6:**
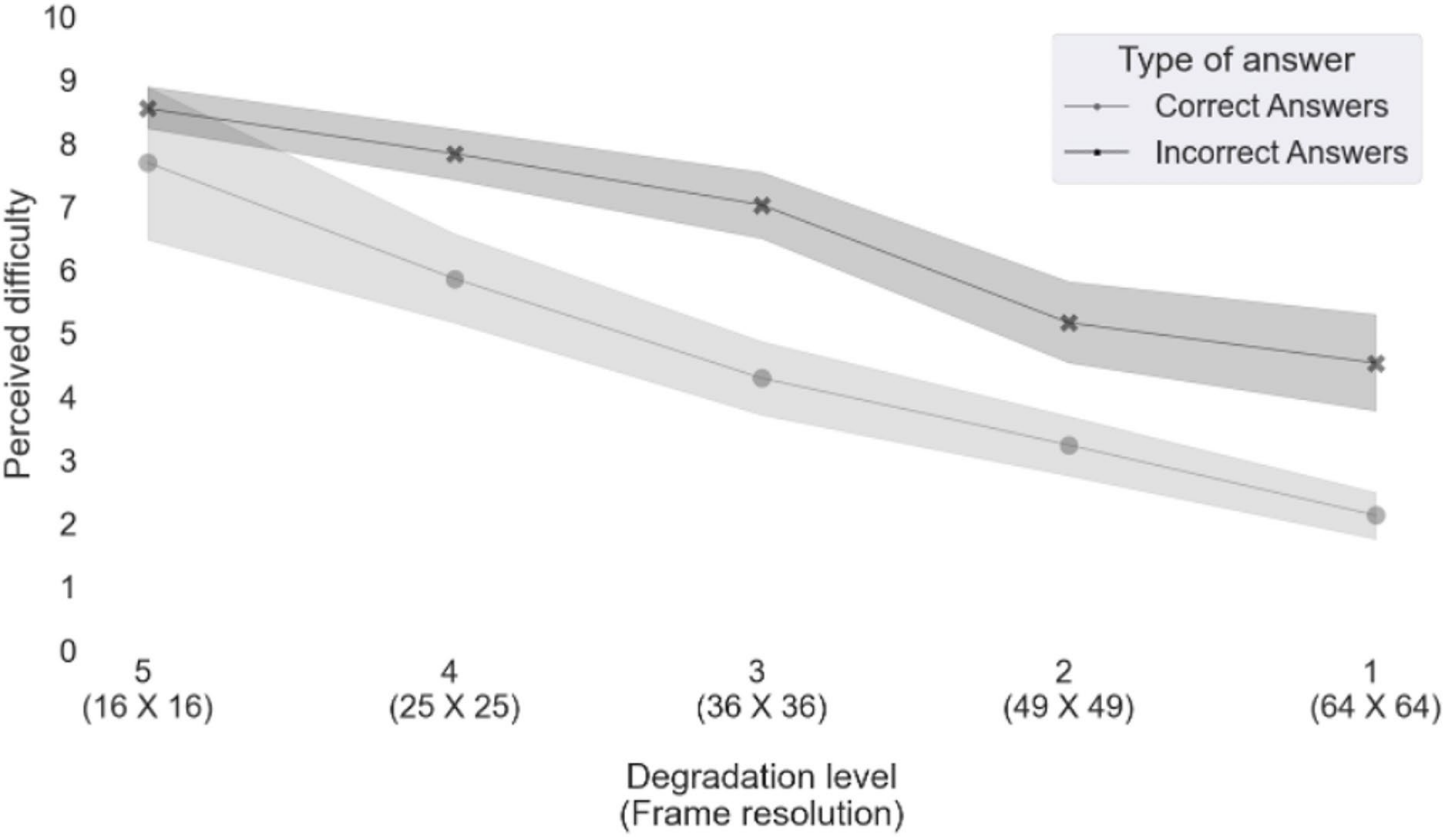
Perceived difficulty ratings separated by correct (light grey dots) and incorrect (dark grey crosses) answers. Error-band represents 95% confidence interval.

There was a significant interaction between response accuracy and the degradation level $\:\beta\:\:$= 1.26, SE = 0.10, z = 2.92, *p*_*BH*_ = 0.007, The pattern of the interaction indicated that, as the degradation level increased, the perceived difficulty increased more for correct than for incorrect answers, with indistinguishable perceived difficulty between incorrect and correct answers for the highest degradation level (see Fig. 6), this matches our associations in the interaction from our main analysis in the spectral degradation condition.

#### Background noise

As in the main analysis, there was a statistically significant effect of level of degradation, $\:\beta\:$ = 1.74, SE = 0.08, z = 11.80, *p*_*BH*_ < 0.001, showing that higher degradation levels were associated with higher perceived difficulty ratings. For response accuracy, correct answers showed lower difficulty rating than incorrect answers, $\:\beta\:$ = 0.38 SE = 0.03, z = -11.88, *p*_*BH*_ < 0.001. The results indicate that both degradation level and intelligibility have significant effects on perceived difficulty, with lower degradation levels and correct answers being associated with lower difficulty ratings. Trial number had no significant effect on the difficulty ratings ($\:\beta\:$ = 0.95, SE = 0.03, z = -1.44, *p*=.187). The interaction between degradation level and response accuracy was not significant (see Table 4), suggesting that the influence of degradation level on perceived difficulty does not change with intelligibility.

**Table 4:** Results for the general linear mixed model with difficulty rating as outcome variable for background noise. All reported metrics are standardized.

	β	Std. Error	CI	Z-statistic	Pr_BH_ (>|z|)
(Intercept)	1.98	0.30	1.47–2.68	4.45	0.011
Trial number	1.00	0.03	0.93–1.06	0.10	0.917
Degradation level	1.86	0.16	1.57–2.21	7.07	< 0.001
Response accuracy	0.40	0.03	0.34–0.47	-10.88	< 0.001
Degradation level: Response accuracy	1.26	0.10	1.08–1.47	2.92	0.007
Random Effects					
σ^2^	1.82				
τ_00_	0.92 ID_participant_				
	0.09 ID_sign_				
τ_11_	0.08 ID_participant.Degradation level_				
ϱ_01_	-0.65 ID_participant_				
ICC	0.31				
N	28 ID_participant_				
	100 ID_sign_				
Observations	1390				
Marginal R^2 / Conditional R^2	0.2825 / 0.504				

## Discussion

In this study, we designed two types of visually challenging conditions, spectral degradation and background noise, to examine their impact on sign language intelligibility and perceived difficulty. We expected both conditions to negatively impact intelligibility and increase the perceived difficulty. Additionally, we explored the combined effects of degradation levels and response accuracy on perceived difficulty in sign perception. For both conditions, the degradation level and the response accuracy had a significant impact on the perceived difficulty ratings, however, only the spectral degradation condition showed a significant interaction between these factors. Taken together, the findings suggest that sign language intelligibility is sensitive to perceptual degradations of visual signals and that the perceptual processing of these degradations can vary depending on their specific nature.

We defined theoretical psychometric functions for both background noise and spectral degradation, which set the intelligibility level for 50% approximately at our intermediate level of degradation (i.e. slightly below level 3 for background noise and slightly above for spectral degradation). In both conditions, isolated sign intelligibility seems to reach ceiling level approximately at 70% intelligibility. Similar results have been reported for isolated words in background noise for speech (see “words in context,” Fig. 2, p. 121^33^). For spectral degradation, studies suggest that ceiling intelligibility is reached at 8 bands in noise-vocoded isolated words (~ 70–90% depending on the cut-off filter)^34^. This aligns with our ceiling intelligibility performance (72%) found at our 64 × 64 pixel condition. Tachibana and colleages^34^ also assess the effect of 4 bands in word intelligibility and found that performance varied greatly (from ~ 50–80%) depending on the envelope frequency cut-off. While we cannot compare the effect of the cut-off frequency from our study to theirs, our spectral degradation at that level seemed to have a more detrimental effect on intelligibility. Thus, the results seem to suggest that our experimental conditions elicit behavioural performance levels comparable to ceiling effects observed in both speech and sign intelligibility tasks, though more research is needed to assess comparisons at more challenging levels.

Our analyses also showed an increased performance in the spectral degradation condition, but not in the background noise, as the experiment progressed. This draws parallels with previous speech literature theories which describe no habituation for distracting stimuli (e.g., background noise)^35^, and experiments showing a learning effect in spectrally degraded speech^36,37^.

Our exploratory analyses revealed that intelligibility (correct or incorrect) significantly impact on perceived difficulty, even after taking degradation level into account. This suggests that understanding the sign is subjectively associated with less difficulty in identification, even when the quality of the signal does not change. Previous literature has identified that the visual system engages in top-down processes at all stages of the visual identification process and uses contextual cues, such as semantic context, motion and familiarity, to extract relevant information^38^. Future studies would need to assess whether participants are making use of contextual cues to help with sign identification in visually challenging conditions. If so, this would draw another analogy with spoken language. There is evidence in spoken language that familiarity can influence understanding in noise. For example, parameters like lexical frequency and language familiarity impact intelligibility in speech in noise^39,40^: Words that are frequent or highly familiar are easier to recognize in noise, while rare or less familiar words require are more difficult to discern. These aspects were not something we set out to investigate in the present study, but the results from our exploratory analyses suggest that similar effects might occur in sign language processing. This idea should be followed up in future studies.

Our analyses further showed a significant interaction between degradation levels and response accuracy (correct or incorrect), but only for the spectral degradation condition. This interaction could suggest that participants were rating the task in terms of understanding (“how difficult was it to understand?”) instead of perception (“how difficult was it to see?”). However, since this result is not repeated for background noise, this result could suggest that participants were understanding the task correctly, but that the two types of degradation might be engaging different perceptual mechanisms. Specifically, the presence of background noise, requires isolating the relevant signal from competing distractions^35^. It has been established that the visual system can exploit the temporal structure of motion to distinguish objects from noisy backgrounds, which might be the predominant strategy for figure-ground segregation in this case. This recruitment of motion cues engages top-down attentional mechanisms^38^, much like the processing of speech in noise requires top-down mechanisms to discern the relevant signal from the noise^41^. It is likely that this compensatory mechanism is not as critical at lower levels of spectral degradation, where key visual features such as fingers and facial details remain discernible in static frames (see Fig. 2B) in the same way as the envelope of the acoustic signal remains unchanged in auditory spectral degradation^25^. However, as spectral degradation intensifies, specifically after degradation level 3, when the hands and face become fully pixelated, difficulty ratings for correct and incorrect responses begin to converge (see Fig. 6). This result suggests that participants might be shifting their perceptual strategy, thereby changing the trend of the ratings.

Further research is needed to determine whether the observed differences in perceived difficulty reflect distinct perceptual strategies, or involve the engagement of different cognitive mechanisms. If language processing in challenging conditions operates in a modality-independent manner, we would expect background noise to activate frontal lobe regions and involve working memory resources, similar to what has been shown in auditory processing^2^. While there is existing evidence that working memory supports object extraction in visual noise^42^, no comparable studies have investigated this in the context of pixelation. As such, it remains too early to draw firm conclusions about the cognitive demands of spectral degradation and about the modality-independent nature of language processing in perceptually challenging conditions.

This uncertainty about modality-independence was a key motivation behind our methodological approach. Our findings suggest several behavioural parallels between auditory and visual processing in degraded conditions. These observed equivalences might be the result of conditions designed to align the visual degradations with those typically used in speech research. Due to this and given the distinct perceptual mechanisms engaged by background noise and spectral degradation, a methodological discussion is also due, not only to contextualize our design choices, but also to support future replication and comparison.

### Methodological considerations

While we cannot test if the linguistic information was degraded equally across modalities, we designed our spectral degradation be able to draw analogies between the modalities. The degradation levels were designed in a way that each dimension contained as many pixels as the square of a certain value (i.e., $\:{4}^{2},\:{5}^{2},\:{6}^{2},\:{7}^{2}$ and $\:{8}^{2}$, correspond to levels 5 − 1 from the most degraded to the least, respectively ). These values were trying to emulate the common number of bands chosen in vocoded speech (from 4 to 8, from the most degraded to the least, respectively). Another inspiration for our design of the spectral degradation condition is that pixelation has been used to simulate artificial vision in retinal implant research^12,13^, similar to how noise vocoding simulates cochlear implant hearing^25^. Noise-vocoding is designed to reduce information in the temporal domain, where most of the linguistic information is contained^43^. In sign, the signal entropy is contained in the spatial domain^43^, drawing another analogy between both methods of spectral degradation.

For spectral degradation intelligibility, we find a linear trend from levels 5 to 2 and the beginning of a plateau when transitioning from level 2 to 1. A floor performance cannot be estimated from the hardest levels, and the theoretical fit has higher error on the easiest and hardest level. This suggests that both higher and lower difficulties should be added in order to find these levels of intelligibility.

In background noise, the degradation levels were created by substituting a percentage of pixels by salt and pepper noise, creating an effect of superposition with an increase of 1% between each level of degradation. A similar method was used by ^4^, who evaluated intelligibility as a function of the SNRs of the videos. In the same study, they warn researchers that a high SNR can still have low intelligibility if the noise is inconveniently spatially placed. For example, if the hands of the signers become noisy but the rest of the frame is noiseless, the SNR will be high, but the intelligibility is likely to be low. Comparing degradation levels in background noise between the auditory and the visual modality is challenging due to the inequivalence of the signal quality metrics. While the auditory modality is heavily reliant on SNR, the visual modality lacks a robust metric of video quality^44,45^ because of the complexities of the visual signal (e.g., contrast, color, saturation, orientation of the object, etc.). These factors might explain how the performance varied so much with only a 1% difference in noise percentage (see Sect.  3.2.1) when most auditory research studies intelligibility varying the SNR in more than 3 decibels (~ 25% change in noise level) between each degradation level (e.g.^16,33,46,47^).

### Limitations and future studies

All participants in the study were fluent in sign language, however, the variability in language knowledge was not controlled for. Following research on speech perception, it has been suggested that high intelligibility might still require more effort for less knowledgeable participants^39,40^. Linguistic item characteristics, language proficiency and other cognitive metrics (i.e., working memory) should also be assessed to evaluate whether the effects of perceptual impairments are constant across language modalities. In speech, high-frequency words are recognised faster and more accurately than low-frequency words (e.g.^48–50^). Language familiarity and exposure have also been found to play a role in speech intelligibility under different effortful listening conditions^51^, with native speakers having an advantage over non-native speakers and regular speakers over inexperienced users of the language, respectively.

Our study also found evidence suggesting that visually challenging conditions are perceived differently when individuals comprehend the signs. Future studies assessing the effect of sign characteristics and/or participants’ cognitive abilities should be performed to understand how objective levels of visual impairments might be perceived differently depending on context.

Finally, our exploratory analyses suggest that different mechanisms may be involved in extracting information from visually degraded stimuli. Future neuroimaging research is needed to determine whether this reflects distinct visual strategies employed by the system, or whether separate cognitive pathways are recruited depending on the type of visual challenge, paralleling findings in speech perception^2^.

## Conclusion

In this study, two visually challenging conditions (spectral degradation and background noise) were created to assess their impact on sign language intelligibility and perceived difficulty of isolated item identification.

Intelligibility followed a predictable trend, reaching a ceiling around 70% accuracy, similar to studies in the auditory modality in both degraded conditions using isolated words. Both spectral degradation and background noise reduced intelligibility and increased perceived difficulty, but only spectral degradation showed an interaction between degradation level and response accuracy. These findings suggest that the visual system relies on different perceptual mechanisms depending on the nature of the degradation. Our results show similarities to spoken language, as correct answers were rated as easier to see than incorrect ones, and the perceptual mechanisms involved in processing signs under background noise and spectral degradation in the visual domain seem to match those from the auditory domain.

Future research should examine linguistic characteristics, language proficiency, and cognitive factors (e.g., working memory) impact on sign language intelligibility and perceived difficulty under visually challenging conditions. Additional degradation levels could be tested to better estimate floor and ceiling effects. Finally, neuroimaging studies that compare auditory and visual modalities under perceptually challenging conditions could offer deeper insights into the similarities in perceptual processing across modalities.

## Supplementary Information

Below is the link to the electronic supplementary material.

Supplementary Material 1

Supplementary Material 2

Supplementary Material 3

Supplementary Material 4

## Data Availability

All data, including raw and processed datasets, experimental stimuli, analysis scripts, and figure-generation code, are publicly available on the Open Science Framework (OSF) at https://osf.io/yb2nw/files/osfstorage^52^. The repository has been structured to follow FAIR principles (Findable, Accessible, Interoperable, and Reusable) to facilitate transparency, reproducibility, and reuse of the materials..
