# Supplementary material for: Visually challenging conditions on sign language intelligibility show behavioural analogies with spoken language
Source: Sci Rep. 2026 Jun 13;16:18359. doi: 10.1038/s41598-026-57531-0 (PMC13264605; doi:10.1038/s41598-026-57531-0)
Supplement: Supplementary file 1 — Supplementary Material 1 [file 41598_2026_57531_MOESM1_ESM.docx]

Table 1 Linguistic properties of selected signs

| **Filename** | **Swedish Word** | **English Translation** | **Sign Duration (ms)** | **Zipf** | **Phonotactic Probability** | **Neighbourhood Density** |
| --- | --- | --- | --- | --- | --- | --- |
| 1003-R-10864 | hackor | pickaxe | 1040 | 3,11 | 0,28 | 1.95 |
| 1004-R-2817 | du | you | 860 | 6,44 | 0,51 | 1.8 |
| 1008-R-7696 | gång | walk | 1470 | 5,77 | 0,21 | 2.5 |
| 1011-R-17137 | linbana | cable car | 1370 | 2,74 | 0,20 | 2.8 |
| 1012-R-3194 | träffa | meet | 890 | 5,16 | 0,31 | 2.75 |
| 1024-R-11199 | alfabet | alphabet | 860 | 2,65 | 0,21 | 2.45 |
| 1032-R-2531 | kompis | mate | 480 | 4,96 | 0,18 | 1.8 |
| 1048-R-6277 | Herrljunga | Herrljunga | 700 | 2,86 | 0,15 | 3.4 |
| 1056-R-5538 | ärtsoppa | pea soup | 720 | 3,11 | 0,22 | 2.35 |
| 1063-R-1070 | brinna | burn | 1090 | 3,73 | 0,15 | 2.0 |
| 1064-R-8640 | tempo | pace | 880 | 4,28 | 0,27 | 3.65 |
| 1067-R-4401 | hörlur | headphone | 630 | 2,05 | 0,13 | 2.05 |
| 1072-R-2966 | ofta | often | 890 | 5,42 | 0,28 | 2.65 |
| 1076-R-6388 | Lund | Lund | 650 | 4,38 | 0,17 | 2.75 |
| 1079-R-3340 | bli förskräckt | be startled | 800 | 3,21 | 0,12 | 1.8 |
| 1091-R-4686 | Bryssel | Brussels | 870 | 3,81 | 0,17 | 2.25 |
| 1095-R-14792 | sluta | stop | 620 | 5,10 | 0,20 | 1.05 |
| 1096-R-128 | kudde | pillow | 550 | 4,04 | 0,11 | 2.25 |
| 1099-R-7466 | broms | brake | 490 | 3,14 | 0,20 | 2.9 |
| 1104-R-4072 | jävla | damn | 690 | 5,30 | 0,31 | 2.8 |
| 1108-R-1092 | leka | play | 840 | 4,82 | 0,23 | 3.4 |
| 1116-R-7940 | dum | stupid | 580 | 4,51 | 0,19 | 2.35 |
| 1127-R-2246 | cykla | cycle | 1160 | 4,48 | 0,21 | 2.7 |
| 1128-R-832 | tala flytande | speak fluently | 900 | 4,08 | 0,19 | 1.85 |
| 1131-R-12469 | fix | fix | 800 | 4,04 | 0,16 | 4.3 |
| 1135-R-17763 | själva | themselves | 930 | 5,32 | 0,21 | 3.25 |
| 1143-R-18687 | fiska upp någon | fish up someone | 880 | 3,82 | 0,11 | 4.3 |
| 1144-R-12177 | sil | sieve | 980 | 3,02 | 0,07 | 4.5 |
| 1147-R-11502 | detektiv | detective | 670 | 2,52 | 0,24 | 3.65 |
| 1151-R-6806 | frälsa | save | 690 | 2,69 | 0,16 | 4.4 |
| 1167-R-1410 | ficklampa | flashlight | 670 | 3,13 | 0,33 | 5.55 |
| 1175-R-1575 | dra av | pull off | 800 | 5,21 | 0,15 | 2.95 |
| 1176-R-3691 | ta ut | take out | 530 | 6,08 | 0,12 | 2.45 |
| 1195-R-5670 | i tre år | for three years | 990 | 5,60 | 0,22 | 5.0 |
| 1196-R-11833 | varje dag | every day | 660 | 5,67 | 0,16 | 3.5 |
| 1200-R-1399 | faster | aunt | 780 | 4,12 | 0,25 | 2.55 |
| 1208-R-18984 | Norden | North | 630 | 3,83 | 0,15 | 3.35 |
| 1220-R-2025 | knacka | knock | 580 | 3,42 | 0,18 | 3.45 |
| 1223-R-3104 | tål inte varandra | can't stand each other | 710 | 4,12 | 0,15 | 1.65 |
| 1227-R-1401 | entusiastisk | enthusiastic | 710 | 3,05 | 0,21 | 2.6 |
| 1231-R-2073 | slipa | grind | 760 | 3,50 | 0,16 | 4.45 |
| 1232-R-19991 | skriva upp | write up | 910 | 5,52 | 0,16 | 2.75 |
| 1235-R-912 | färg | colour | 600 | 5,12 | 0,19 | 1.75 |
| 1236-R-2694 | säga | say | 490 | 5,71 | 0,31 | 2.55 |
| 1239-R-1238 | svarslös | unresponsive | 480 | 2,18 | 0,17 | 2.65 |
| 1240-R-14984 | krocka | crash | 550 | 3,17 | 0,20 | 1.95 |
| 1244-R-11625 | subjekt | subject | 620 | 2,50 | 0,26 | 3.75 |
| 1252-R-9073 | överflödig | redundant | 1820 | 3,18 | 0,24 | 4.8 |
| 1255-R-1404 | certifikat | certificate | 1020 | 2,69 | 0,12 | 4.6 |
| 1256-R-677 | skidor | ski | 900 | 4,11 | 0,26 | 3.55 |
| 1260-R-12457 | magi | magic | 830 | 3,59 | 0,18 | 5.65 |
| 1271-R-10038 | känslig | sensitive | 930 | 4,18 | 0,19 | 5.0 |
| 1276-R-1056 | tvätta | wash | 1080 | 4,73 | 0,24 | 2.45 |
| 1284-R-6912 | jul | Christmas | 1090 | 5,03 | 0,21 | 2.85 |
| 1288-R-3370 | Polen | Poland | 1090 | 3,96 | 0,20 | 2.65 |
| 1291-R-2533 | spärra | block | 1220 | 3,12 | 0,21 | 2.0 |
| 1296-R-13485 | volt | volt | 1300 | 3,58 | 0,20 | 1.75 |
| 1299-R-1022 | reagera | react | 860 | 4,02 | 0,20 | 1.55 |
| 1307-R-2839 | lördag | Saturday | 970 | 5,16 | 0,39 | 1.65 |
| 1308-R-11129 | syd | south | 780 | 3,43 | 0,32 | 1.75 |
| 1315-R-15829 | ta emot | receive | 840 | 5,29 | 0,14 | 3.45 |
| 1324-R-16236 | urval | selection | 1080 | 3,76 | 0,16 | 3.9 |
| 1327-R-2108 | få en stämpel på sig | get a stamp on it | 890 | 3,27 | 0,13 | 3.4 |
| 1331-R-298 | före | before | 1220 | 5,08 | 0,21 | 3.2 |
| 1332-R-27 | roa sig | enjoy | 920 | 3,96 | 0,18 | 3.1 |
| 1335-R-17064 | bit | bit | 1230 | 4,86 | 0,12 | 2.7 |
| 1336-R-3010 | tid | time | 770 | 5,74 | 0,24 | 2.1 |
| 1339-R-5236 | torka | wipe | 1070 | 4,35 | 0,15 | 2.2 |
| 1355-R-7867 | nära | close | 930 | 5,14 | 0,16 | 4.7 |
| 1368-R-11900 | tjugotvå | twenty-two | 980 | 2,68 | 0,25 | 5.55 |
| 1371-R-5533 | flera | several | 930 | 5,51 | 0,29 | 2.45 |
| 1372-R-207 | vänta | wait | 960 | 5,08 | 0,32 | 1.4 |
| 1379-R-371 | avdelning | department | 1230 | 3,90 | 0,21 | 3.0 |
| 1380-R-17381 | påhittad | imaginary | 1060 | 3,04 | 0,16 | 3.8 |
| 1384-R-1660 | tiger | tiger | 1090 | 4,12 | 0,13 | 2.8 |
| 1392-R-4789 | bli mindre | become smaller | 870 | 5,41 | 0,24 | 2.65 |
| 1396-R-3351 | läppstift | lipstick | 1180 | 4,14 | 0,23 | 1.4 |
| 1404-R-15724 | ritual | ritual | 1160 | 2,95 | 0,17 | 1.95 |
| 1407-R-3523 | inställning | setting | 1090 | 4,24 | 0,14 | 4.75 |
| 1408-R-4907 | internet | internet | 1320 | 4,98 | 0,17 | 5.5 |
| 1415-R-4567 | fredag | Friday | 890 | 5,25 | 0,28 | 2.35 |
| 1416-R-19469 | Tromsö | Tromso | 1170 | 2,58 | 0,18 | 3.6 |
| 1423-R-4291 | för länge sedan | long ago | 870 | 5,64 | 0,25 | 3.8 |
| 1435-R-17227 | Babblarna | Babblarna | 1260 | 2,80 | 0,25 | 2.05 |
| 1436-R-262 | område | area | 1340 | 4,34 | 0,33 | 1.9 |
| 1451-R-8230 | frisyr | hairstyle | 1410 | 4,19 | 0,13 | 4.95 |
| 1452-R-9989 | euro | euro | 900 | 4,11 | 0,17 | 2.75 |
| 1456-R-11827 | tveka | hesitate | 1300 | 3,82 | 0,25 | 2.0 |
| 1459-R-228 | hugga | chop | 940 | 3,70 | 0,21 | 2.4 |
| 1460-R-4043 | avstå | refrain | 930 | 3,79 | 0,08 | 4.85 |
| 1463-R-247 | hög | high | 890 | 4,97 | 0,30 | 1.25 |
| 1467-R-1076 | hjälm | helmet | 820 | 3,92 | 0,17 | 3.55 |
| 1471-R-4123 | röka | smoke | 920 | 4,10 | 0,22 | 2.6 |
| 1476-R-11603 | dregla | drool | 900 | 3,05 | 0,16 | 2.9 |
| 1480-R-1249 | puck | puck | 1320 | 3,34 | 0,11 | 3.5 |
| 1487-R-2041 | verkstad | workshop | 990 | 3,50 | 0,13 | 6.25 |
| 1488-R-17022 | från och med nu | from now on | 1000 | 6,45 | 0,18 | 1.95 |
| 1492-R-10997 | fälla en tår | shed a tear | 810 | 3,92 | 0,30 | 1.65 |
| 1507-R-2616 | föredra | prefer | 970 | 3,62 | 0,21 | 3.0 |
| 1516-R-18766 | Celsius | Celsius | 960 | 3,62 | 0,15 | 2.4 |
| 1571-R-9137 | ösregna | pouring rain | 1260 | 2,88 | 0,15 | 2.65 |
| 1592-R-7104 | reinkarnation | reincarnation | 2200 | 2,54 | 0,13 | 5.3 |
| 1899-R-80 | fred | peace | 1050 | 4,30 | 0,24 | 1.85 |
| 2023-R-3410 | skriva | writing | 1070 | 5,52 | 0,23 | 1.9 |
