# Supplementary figures and images for: Visually challenging conditions on sign language intelligibility show behavioural analogies with spoken language
Source: Sci Rep. 2026 Jun 13;16:18359. doi: 10.1038/s41598-026-57531-0 (PMC13264605; doi:10.1038/s41598-026-57531-0)

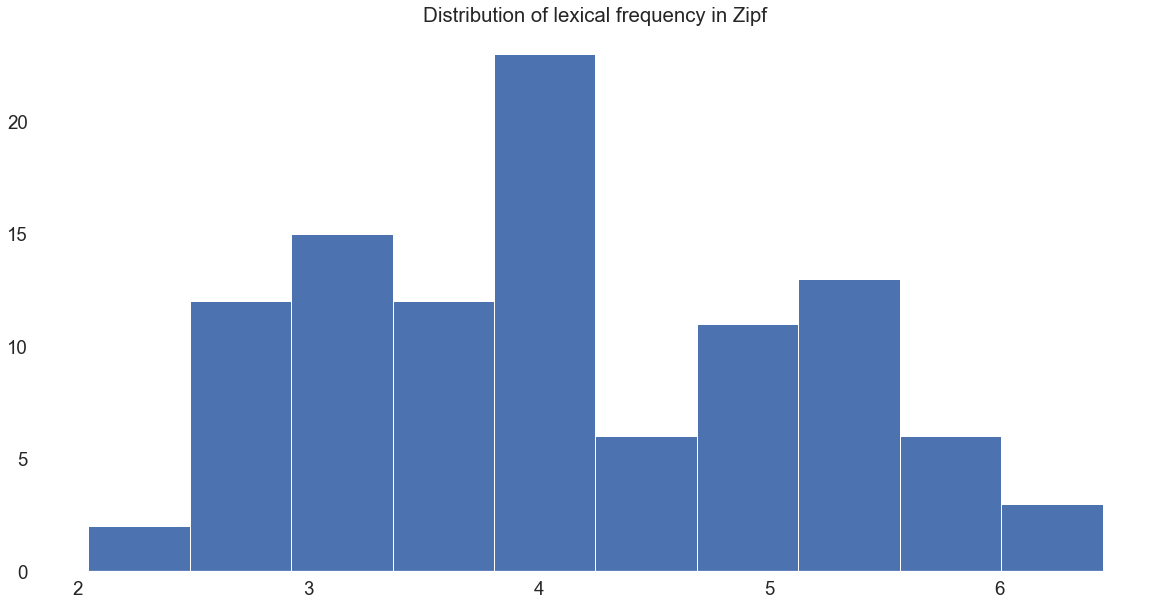

Supplement: Supplementary file 2 — Supplementary Material 2 [file 41598_2026_57531_MOESM2_ESM.png]

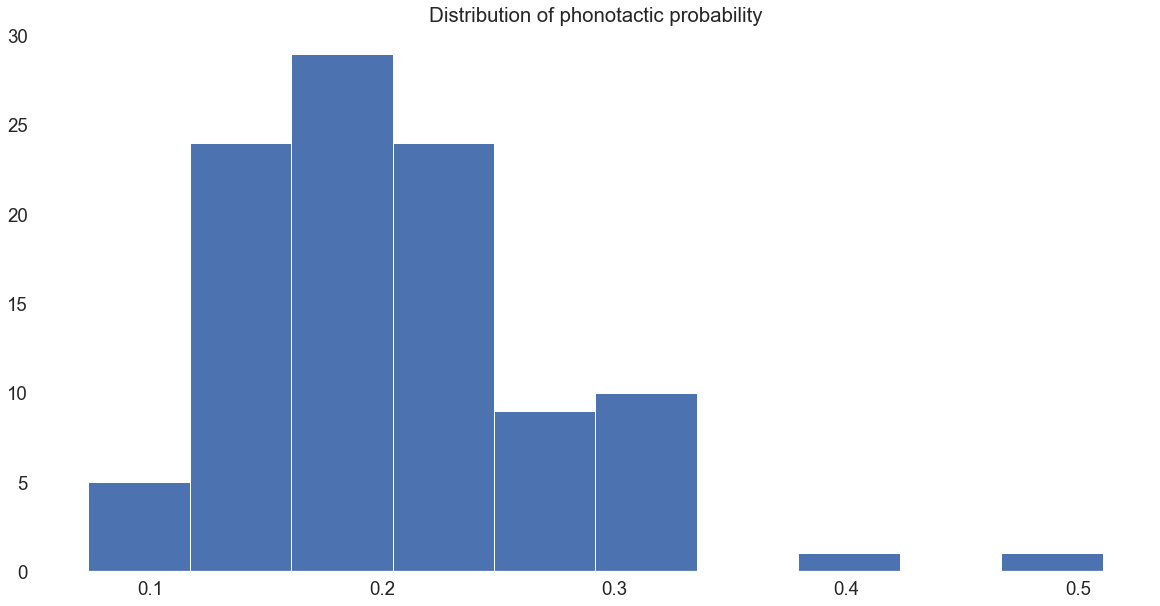

Supplement: Supplementary file 3 — Supplementary Material 3 [file 41598_2026_57531_MOESM3_ESM.png]

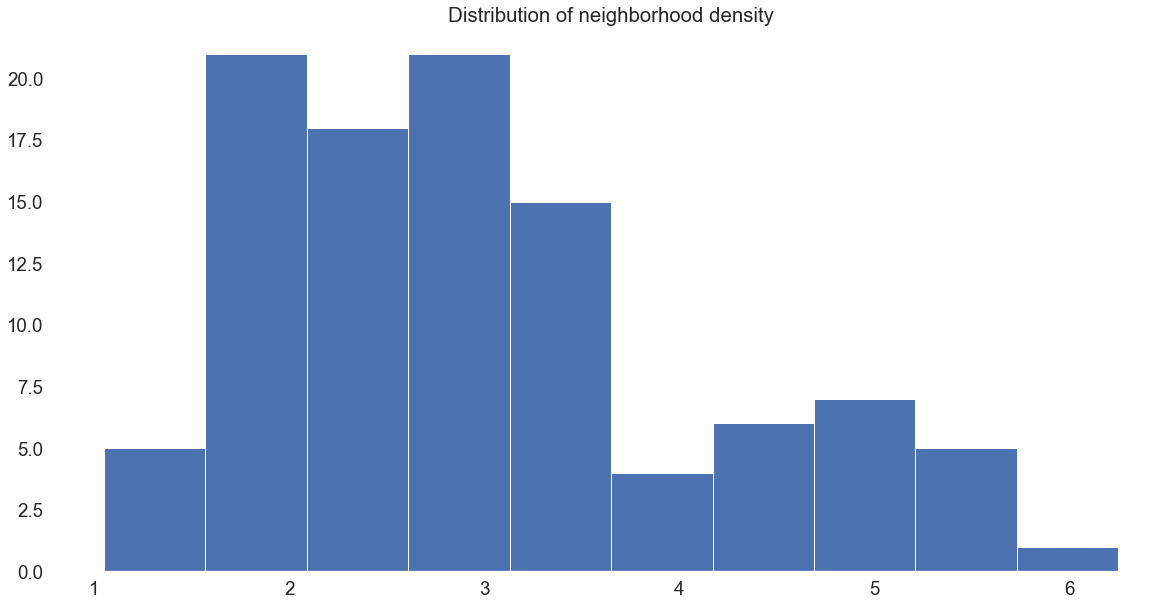

Supplement: Supplementary file 4 — Supplementary Material 4 [file 41598_2026_57531_MOESM4_ESM.png]
